# Emerging Strategies in Mesenchymal Stem Cell-Based Cardiovascular Therapeutics

**DOI:** 10.3390/cells13100855

**Published:** 2024-05-17

**Authors:** Rishabh Kumar, Nitin Mishra, Talan Tran, Munish Kumar, Sivakumar Vijayaraghavalu, Narasimman Gurusamy

**Affiliations:** 1 Department of Biochemistry, Faculty of Science, University of Allahabad, Prayagraj 211002, India; 2 Department of Pharmaceutical Sciences, Barry and Judy Silverman College of Pharmacy, Nova Southeastern University, 3200 South University Drive, Fort Lauderdale, FL 33328-2018, USA; 3 Department of Life Sciences, Manipur University, Imphal 795003, India; drshiva@manipuruniv.ac.in

**Keywords:** mesenchymal stem cells (MSCs), cardiovascular therapeutics, cell-based therapy, regenerative medicine, extracellular vesicles

## Abstract

Cardiovascular diseases continue to challenge global health, demanding innovative therapeutic solutions. This review delves into the transformative role of mesenchymal stem cells (MSCs) in advancing cardiovascular therapeutics. Beginning with a historical perspective, we trace the development of stem cell research related to cardiovascular diseases, highlighting foundational therapeutic approaches and the evolution of cell-based treatments. Recognizing the inherent challenges of MSC-based cardiovascular therapeutics, which range from understanding the pro-reparative activity of MSCs to tailoring patient-specific treatments, we emphasize the need to refine the pro-regenerative capacity of these cells. Crucially, our focus then shifts to the strategies of the fourth generation of cell-based therapies: leveraging the secretomic prowess of MSCs, particularly the role of extracellular vesicles; integrating biocompatible scaffolds and artificial sheets to amplify MSCs’ potential; adopting three-dimensional ex vivo propagation tailored to specific tissue niches; harnessing the promise of genetic modifications for targeted tissue repair; and institutionalizing good manufacturing practice protocols to ensure therapeutic safety and efficacy. We conclude with reflections on these advancements, envisaging a future landscape redefined by MSCs in cardiovascular regeneration. This review offers both a consolidation of our current understanding and a view toward imminent therapeutic horizons.

## 1. Introduction

Cardiovascular diseases remain leading causes of morbidity and mortality worldwide, presenting an unrelenting challenge to the medical community [1]. In recent decades, traditional treatments, including pharmaceuticals, lifestyle modifications, and surgical interventions, have undeniably advanced. However, the complex nature of cardiovascular diseases, which are characterized by damage to the myocardium, limited regenerative potential, and progressive heart failure, demands innovative therapeutic approaches [2]. Stem cells, with their intrinsic ability to self-renew and differentiate into various cell types, have presented a promising avenue for cardiovascular regeneration [3]. The initial optimism surrounding stem cell-based therapies stemmed from the prospect of regenerating damaged myocardial tissue, thereby potentially reversing the effects of conditions such as myocardial infarction [3].

Mesenchymal stem cells (MSCs) have been at the forefront of this research, primarily due to their multilineage differentiation potential, immunomodulatory properties, and relative ease of isolation from various tissues [4]. Early studies focused on the direct transplantation of MSCs into damaged cardiac tissue, with the aim of replacing lost cardiomyocytes and restoring heart function [4]. However, as the field evolved, it became evident that the therapeutic benefits of MSCs extended beyond mere cell replacement. MSCs were found to play a pivotal role in modulating the cardiac microenvironment through paracrine signaling, thus aiding in tissue repair and reducing inflammation [4]. The next phase of research shifted toward understanding these intricate mechanisms, paving the way for the second and third generations of cell-based therapies, which focused on enhancing the efficacy and delivery of stem cell-derived factors [5]. Today, as we stand at the precipice of the fourth generation of stem cell-based therapies, the emphasis is on harnessing the full therapeutic potential of MSCs, fine-tuning their properties, and developing innovative strategies to address the multifaceted challenges of cardiovascular diseases.

To conduct a thorough review of MSC-based cardiovascular therapeutics, we systematically selected and analyzed relevant articles using databases like PubMed, Scopus, and Web of Science. Our search was guided by specific keywords related to MSCs, cardiovascular diseases, and stem cell therapies. We included the most recent peer-reviewed articles, reviews, and clinical trial reports, focusing on their relevance, innovation, and contributions to the field. Emphasis was placed on articles detailing emerging technologies in tissue engineering, genetic modifications of MSCs, and cell-free therapies. Each article was critically evaluated for methodological soundness and its implications for future research and clinical applications, ensuring a comprehensive overview of the latest advancements and gaps in MSC-based cardiovascular therapies.

## 2. Past and Present Stem Cell-Based Therapeutic Approaches

The historical journey of stem cell therapies in cardiovascular medicine began when scientists explored bone marrow transplantation for the treatment of blood disorders [6]. The discovery of embryonic stem cells toward the end of the 20th century opened up new possibilities for regenerative medicine.

Among the earliest and most significant developments was the application of bone marrow-derived stem cells for myocardial repair. Pioneering studies such as the BOOST trial [7] underscored the potential of bone marrow-derived mononuclear cells to enhance left ventricular ejection fraction following myocardial infarction. Despite these promising results, subsequent larger trials like REPAIR-AMI presented a more nuanced picture, showing modest improvements in specific patient subsets and raising questions about the efficacy and applicability of these therapies [8,9]. Embryonic stem cells, known for their pluripotency, emerged as a promising candidate in cardiovascular therapeutics [10]. However, their use was fraught with ethical concerns, especially regarding the use of embryonic material [11]. Additionally, the risk of teratoma formation and immunological complications associated with embryonic stem cells posed significant hurdles to their direct clinical application [12]. These challenges necessitated a shift toward more ethically acceptable and patient-specific approaches.

### 2.1. Induced Pluripotent Stem Cells (iPSCs)

The development of induced pluripotent stem cells (iPSCs) by Shinya Yamanaka in 2006 significantly advanced stem cell research, offering a potent alternative to embryonic stem cells without associated ethical concerns [13]. iPSCs are reprogrammed from adult cells to a pluripotent state, facilitating the generation of patient-specific cells that bypass immunological issues inherent in embryonic sources [14]. iPSC-derived cardiomyocytes have emerged as crucial tools in drug testing, disease modeling, and cardiac therapy [15,16]. Due to their ability to differentiate into cardiomyocytes, iPSCs have opened new pathways for cardiac repair and regeneration, showcasing their utility in personalized medicine for designing patient-specific treatments [17]. Moreover, iPSC-derived cardiac cells have been instrumental in advancing our understanding of cardiac diseases at a molecular level, enhancing disease modeling and fostering the development of targeted therapies [18,19].

To enhance the efficacy, safety, and delivery of iPSC-based therapies for cardiovascular applications, several innovative approaches are being pursued. (a) Three-Dimensional bioprinting: This technology that constructs functional tissues that mimic natural cardiovascular structures, allowing for the precise placement of iPSC-derived cells in a biomaterial matrix. It enhances cell maturation, integration, and function in a controlled environment [20]. (b) Tissue engineering: Techniques are developed to create viable cardiac tissue grafts from iPSCs, using biodegradable scaffolds that support cell growth and integration. These tissues can replace a damaged myocardium and incorporate features like electrical conductivity to boost functional integration [21,22]. (c) Genetic modifications: iPSCs are genetically modified to improve survival, proliferation, and differentiation. Techniques like CRISPR/Cas9 are employed to enhance cellular properties and reduce sources of risk such as immunogenicity [23]. (d) Delivery mechanisms: Innovative delivery methods like catheter-based injections, magnetic targeting, and microcarriers are used to enhance the precision and retention of iPSC-derived cells in the myocardium [24]. Despite progress, challenges like ensuring long-term cell survival, preventing immune rejection, and adhering to regulatory standards remain [24]. Ongoing research aims to improve safety profiles and optimize cellular functions, moving toward more effective, safe, and customized iPSC-based cardiovascular therapies [25].

### 2.2. Cardiosphere-Derived Cells

Cardiosphere-derived cells (CDCs) are sourced from cardiac biopsies and hold significant potential for cardiac regeneration, bridging traits between MSCs and cardiac progenitor cells [26]. These cells have been integral in promoting angiogenesis, reducing fibrosis, and modulating immune responses in post-myocardial infarction scenarios, with their paracrine effects, particularly through the secretion of exosomes and microRNAs, playing pivotal roles in cardiac repair [26]. The CADUCEUS trial notably demonstrated the safety and effectiveness of CDCs in myocardial infarction patients by significantly reducing scar mass and increasing viable heart tissue, highlighting their potential in enhancing myocardial regeneration [8]. Apart from CDCs, other types of cardiac progenitor cells have been explored for their regenerative potential. A study by Mishra et al. [10] demonstrated the therapeutic potential of a unique population of cardiac progenitor cells identified by their expression of Islet-1 in repairing a damaged myocardium. These cells were found to contribute to the formation of new cardiac muscle and blood vessels in animal models, opening new avenues for clinical applications in human heart repair [10].

### 2.3. Endothelial Progenitor Cells

The angiogenic capabilities of endothelial progenitor cells (EPCs) are essential to restoring vascular health [27]. Derived from bone marrow, EPCs repair and maintain the endothelial lining and are crucial for treating cardiovascular diseases marked by endothelial dysfunction [27]. They express specific markers such as CD34, VEGFR2, and CD133, which facilitate their identification and differentiation within the vascular system [28]. Therapeutically, EPCs are utilized in novel treatments like EPC-capture stents, which enhance vascular healing by attracting EPCs to damaged sites, thereby promoting endothelialization and reducing stent thrombosis risks [29]. Additionally, optimizing EPC mobilization and homing—which are affected by physical activity, oxidative stress, and pharmacological interventions—is a vibrant research area, pushing forward cell-based therapies that promise new treatments for complex cardiovascular ailments [30].

### 2.4. MSCs

MSCs are multipotent stromal cells capable of differentiating into a variety of cell types, such as osteoblasts, chondrocytes, myocytes, and adipocytes [31]. Originating from sources including bone marrow, adipose tissue, and umbilical cord blood, MSCs are characterized by their ability to self-renew, minimal immunogenicity, and pronounced immunomodulatory properties, making them particularly valuable for regenerative medicine applications, notably in cardiovascular therapeutics [31]. In the realm of cardiovascular disease treatment, MSCs offer several advantageous properties: they modulate immune responses to reduce inflammation; attenuate fibrosis, which can impair cardiac function; promote angiogenesis, which is necessary for tissue repair and improved blood circulation; and potentially differentiate into cardiomyocyte-like cells to directly aid cardiac repair [32]. While preclinical studies affirm MSCs’ efficacy in enhancing cardiac function post myocardial infarction through engraftment and the facilitation of cardiac repair, their clinical utility is tempered by challenges such as the low retention rate and survival of transplanted cells within the ischemic myocardial environment [33,34,35,36,37,38]. In recent years, focus has shifted toward optimizing the therapeutic potential of stem cells. This involves ensuring better cell survival post transplantation, enhancing the cells’ reparative properties, understanding patient-specific factors, and developing combinatorial therapies involving scaffolds, exosomes, and tissue engineering [39].

Ongoing research and clinical trials continue to explore MSCs’ therapeutic potential, underscoring their significant prospective impact on the treatment of myocardial infarction and other cardiac ailments. The MSC-HF Trial, a randomized, double-blind, placebo-controlled study, evaluated the 4-year outcomes of intramyocardial injections of autologous bone marrow-derived MSCs in patients with ischemic heart failure [40]. It included 60 patients with significant heart failure, showing that MSC treatment led to significant improvements in left ventricular end-systolic volume, ejection fraction, stroke volume, and myocardial mass over 12 months, along with reduced scar tissue and enhanced quality of life [40]. After four years, the data indicated fewer hospitalizations for angina, with no adverse effects noted [40]. The C-CURE Trial assessed the safety and efficacy of MSCs treated with a cardiogenic cocktail in chronic heart failure patients [41]. This multicenter, randomized trial showed improvements in cardiac function, 6-minute walk distance, and composite clinical scores, underscoring the potential of cardiopoietic stem cell therapy [41]. The CHART-1 Trial, another randomized, double-blind study, did not meet its primary efficacy endpoint but revealed potential benefits in specific patient subgroups with certain baseline ventricular volumes, pointing to the possibility of targeted therapy [42]. The ongoing DREAM-HF Trial, the largest among the reviewed studies, is examining the efficacy and safety of mesenchymal precursor cells in advanced chronic heart failure [43]. Although not all primary endpoints were met, there were indications of reduced hospital readmissions and improved cardiac function in certain subgroups, highlighting the nuanced potential of MSC therapies in heart failure [43]. These studies collectively advance the understanding of MSC-based therapies in cardiac care, showing significant promise and guiding future research toward more targeted and personalized approaches. 

### 2.5. MSC-Derived Extracellular Vesicles

Extracellular vesicles (EVs) derived from MSCs have emerged as promising therapeutic agents in the field of cardiovascular diseases [44,45]. EVs, encompassing exosomes and microvesicles, are nanosized lipid bilayer-enclosed structures capable of transferring proteins, lipids, and nucleic acids between cells, thereby modulating cellular functions and responses [45]. In the context of cardiovascular diseases, MSC-derived EVs exhibit potential in enhancing angiogenesis, reducing fibrosis, and modulating inflammatory responses, which are crucial for myocardial repair and regeneration [46]. The therapeutic efficacy of EVs is largely attributed to their ability to mirror the regenerative and reparative properties of their parent MSCs while offering an advantage in terms of safety and feasibility as they lack the risks associated with stem cell implantation, such as tumorigenicity and immune rejection [47]. This study has investigated the use of cardiomyocyte-targeting exosomes loaded with microRNA-302 (miR302) for treating myocardial ischemia/reperfusion injury, finding that these engineered exosomes enhanced cardiomyocyte proliferation and activity and reduced heart damage in vitro and in mice [47]. Recent advances have focused on optimizing the isolation, characterization, and scalability of EV production under good manufacturing practice (GMP) conditions [48]. Furthermore, research is exploring the potential of engineering EVs to enhance their targeting efficiency and therapeutic payload, making them a highly versatile and promising tool in regenerative medicine for cardiovascular therapies [48].

## 3. Biological Properties of MSCs

The therapeutic potential of MSCs in cardiovascular diseases derives from their unique biological properties and actions, which are essential for tissue repair, and researchers are seeking insights into optimizing their clinical use. This section details the key attributes and mechanisms that make them suitable for cardiovascular therapies.

### 3.1. Multipotency and Differentiation

MSCs have the ability to differentiate into cardiomyocytes, endothelial cells, and smooth muscle cells, key components in cardiac tissue repair and vascular regeneration [31]. For MSCs, the process of differentiating into these lineages is governed by a complex interplay of signaling pathways and environmental cues. For instance, the transformation of MSCs into cardiomyocytes often involves the modulation of pathways like Wnt/β-catenin, JAK/STAT, and transforming growth factor-beta (TGF-β) [49,50,51]. A study by Quevedo et al. [52] highlights how MSCs can be induced to differentiate into cardiomyocyte-like cells, subsequently integrating into cardiac tissue and contributing to the restoration of myocardial function in heart failure models. MSC differentiation into endothelial cells, which are essential for angiogenesis and vascular repair, is typically regulated by factors such as vascular endothelial growth factor (VEGF), fibroblast growth factor (FGF)-2, and TGF-β, which activate endothelial markers like von Willebrand factor (vWF), PECAM-1, and vascular endothelial cadherin [53,54]. Similarly, MSC differentiation into smooth muscle cells involves cues from the TGF-β superfamily and PDGF-BB, promoting the expression of smooth muscle markers like α-SMA and calponin [55,56].

Further studies [57] have shed light on the molecular underpinnings of these differentiation pathways. These insights are crucial for developing targeted therapies. For example, understanding the specific signaling molecules and transcription factors, such as stromal-derived factor-1 (SDF-1) and bone morphogenic protein-2, involved in MSC differentiation can lead to the development of biomaterials or scaffold-based approaches that mimic the natural niches of these cells, thereby enhancing their differentiation efficiency and therapeutic efficacy in situ [58]. Moreover, genetic engineering techniques, such as CRISPR/Cas9 and RNA interference, are being explored for the manipulation of these molecular pathways, enhancing the precision of and control over MSC differentiation [59]. The following studies present significant advancements in the field of MSC differentiation and therapeutic applications facilitated by CRISPR/Cas9 technology. Shahabipour et al. [60] developed a CRISPR/Cas9-mediated strategy to insert a DMP1 promoter-driven GFP-DsRed reporter into MSCs, providing a real-time indicator of osteoblast differentiation. Another study [61] explored the role of extracellular vesicles from human-induced pluripotent stem cell-derived MSCs in protecting against renal ischemia–reperfusion injury, highlighting a novel anti-necroptosis mechanism mediated by SP1 delivery and subsequent sphingosine kinase 1 activation. Meshitsuka et al. [62] demonstrated that the CRISPR/Cas9 and AAV-mediated insertion of a B2 microglobulin-HLA-G fusion gene into MSCs could prevent allogeneic rejection, enhancing their utility in off-the-shelf cell therapies. MSC genome editing through a CRISPR-Cas9 ribonucleoprotein delivery method reduced cytotoxicity and enhanced their therapeutic potential [63]. Collectively, these studies underscore the versatility of CRISPR/Cas9 in enhancing MSC applications through precise genetic modifications and functional enhancements.

### 3.2. Immunomodulation

Immunomodulation by MSCs involves the attenuation of inflammation and disease progression [64]. MSCs can interact with various components of the immune system, modulating their activity and thereby reducing inflammation and promoting tissue repair [65]. The immunomodulatory function of MSCs is multifaceted, involving both the secretion of soluble factors and direct cell-to-cell interactions [32]. These cells can secrete a wide range of cytokines, chemokines, and growth factors, such as TGF-β, PGE2, IL-10, and hepatocyte growth factor (HGF), which collectively contribute to the suppression of pro-inflammatory responses and the promotion of anti-inflammatory environments [64]. This secretome alters the behavior of various immune cells, including T cells, B cells, natural killer cells, and dendritic cells, leading to a reduction in inflammatory cytokine production and the suppression of T cell proliferation and cytotoxicity [66].

Prockop and Oh [67] described MSCs as “guardians of inflammation”, highlighting their role in sensing and suppressing excessive inflammatory responses. This description underscores the dynamic and responsive nature of MSCs in modulating the immune response, adapting to the specific inflammatory milieu they encounter. TGF-β contributes to the attenuation of T lymphocyte proliferation by inducing G1 cell cycle arrest through the Jak-1/Stat-5 signaling pathway [68]. MSC-derived EVs deliver thrombospondin 1 (TSP1), which can suppress NK cell activity by modulating TGF-β/Smad signaling [69]. Shi et al. [70] elucidated interactions between MSCs and immune cells, such as the induction of regulatory T cells (Tregs) and the alteration of macrophage phenotypes from a pro-inflammatory (M1) to a regenerative and anti-inflammatory (M2) state. This shift is crucial in mitigating chronic inflammation and facilitating the healing process in cardiovascular tissues.

Furthermore, MSCs can exert immunomodulatory effects through direct cell-to-cell contact involving various adhesion molecules and interactions with immune cell receptors [66]. This contact-dependent mechanism is particularly important in the context of MSCs’ interactions with T cells and antigen-presenting cells, where it can lead to anergy or tolerance, further contributing to a reduction in inflammation [66]. The ability of MSCs to modulate the immune response is not only pivotal in treating inflammatory cardiovascular conditions but also enhances the compatibility and success of other stem cell-based therapies [32]. By creating a more conducive environment for tissue repair and regeneration, MSCs’ immunomodulatory properties can be leveraged to improve outcomes in a wide range of cardiovascular interventions [32].

### 3.3. Paracrine Signaling

MSCs secrete a diverse array of bioactive molecules, including growth factors, cytokines, and extracellular vesicles, which interact with surrounding cells and tissues to induce regenerative processes [71]. MSCs produce growth factors such as VEGF, HGF, insulin-like growth factor (IGF), and platelet-derived growth factor (PDGF) [72]. These factors play critical roles in promoting angiogenesis, the formation of new blood vessels, which is essential for repairing ischemic heart tissues. Angiogenesis facilitates the delivery of oxygen and nutrients to damaged areas, thereby supporting tissue regeneration [72]. Additionally, MSCs secrete cytokines and chemokines that modulate the local immune environment. These include anti-inflammatory cytokines like interleukin-10 (IL-10) and TGF-β, which help mitigate inflammation and fibrosis in the heart, conditions commonly associated with various cardiovascular pathologies [32]. Harrell et al. [73] provide deeper insights into the specific paracrine factors secreted by MSCs and their effects on cardiac tissues.

### 3.4. Extracellular Vesicles and Secretome

MSC-derived EVs, encompassing exosomes and microvesicles, serve as critical conveyors of bioactive molecules including microRNAs, proteins, and lipids that can exert profound influences on recipient cells and tissues [74]. EVs from MSCs are equipped with a diverse array of signaling molecules such as microRNAs that can regulate gene expression in recipient cells, leading to altered cellular behavior such as enhanced survival, reduced apoptosis, and increased angiogenic potential [74]. This capacity to modulate gene expression is crucial in orchestrating tissue repair mechanisms, especially in an ischemic and injured myocardium where the restoration of vascular supply and cellular function is essential [74].

Sahoo et al. [75] demonstrated the proangiogenic activity of EVs derived from human CD34+ stem cells, highlighting their potential in promoting the formation of new blood vessels in damaged cardiac tissue. This angiogenic property is particularly valuable in post-myocardial infarction scenarios in which the restoration of blood flow to ischemic areas is a key factor in limiting infarct size and preserving heart function. Further research by Phinney and Pittenger [76] and Baglio et al. [77] delved into the complex composition of these vesicles. Their studies elucidated the diverse range of proteins, lipids, and nucleic acids present in EVs, each contributing to their regenerative potential. For example, certain proteins within MSC-derived EVs are known to activate signaling pathways involved in cell survival and proliferation, while specific lipids may play a role in membrane interactions and fusion with target cells.

These studies also emphasize the role of the MSC secretome, which includes not only EVs but also soluble factors such as cytokines and growth factors. This secretome can modulate the immune response, reduce inflammation, and enhance the regenerative capacity of heart tissue by recruiting and activating resident cardiac progenitor cells. Understanding the molecular constituents of MSC-derived EVs and their secretome, and how these constituents interact with and influence cardiac cells, opens up novel avenues for therapeutic interventions. By harnessing and potentially engineering these vesicles, it is possible to develop targeted therapies that deliver specific regenerative molecules directly to the site of injury, thereby enhancing the repair and regeneration of cardiac tissues in a more efficient and controlled manner.

Recent studies have highlighted the specific role of miRNAs in modulating the functions and therapeutic potential of MSCs. Zheng et al. [78] found that exosomal miR-9-5p from iPSC-derived MSCs mitigates doxorubicin-induced cardiomyopathy by preventing cardiomyocyte senescence, primarily through the inhibition of the VPO1/ERK signaling pathway. Human umbilical cord-derived MSCs alleviated myocardial fibrosis and restored miRNA-133a expression in diabetic cardiomyopathy, which positively influenced fibrosis markers and inflammatory mediators in a diabetic mouse model [79]. Additionally, the immunoregulatory properties of exosomal miRNAs from bone marrow MSCs overexpressing IDO1 demonstrated their potential to modulate immune responses and improve allogeneic heart transplantation outcomes by influencing key immune-related proteins and miRNAs [80]. Zhu et al. [81] reported that suppressing miR-873-5p rejuvenates aging MSCs, enhancing their functionality and therapeutic efficacy for myocardial infarction repair through modulating autophagy via the AMPK signaling pathway.

In addition to the differentiation of MSCs and the secretion of paracrine factors, MSCs can facilitate cardiac repair through the transfer of mitochondria to damaged cardiac cells [38,82]. This organelle transfer can help rescue injured cardiomyocytes, improve their functional performance, and enhance cell survival under stress conditions [82]. This mechanism could be pivotal in cardiac repair as it directly addresses cellular energy deficits encountered post-injury [82]. Further, MSCs can stimulate the regeneration of endogenous cardiomyocytes through the activation and recruitment of local cardiac stem cells, which can then differentiate and replace damaged myocardial tissue [37,83].

### 3.5. Angiogenesis

MSCs promote the formation of new blood vessels and help restore blood supply to ischemic or damaged heart tissue [84]. They achieve this primarily through the secretion of angiogenic factors such as VEGF, FGF, and HGF [84]. These factors stimulate the proliferation and migration of endothelial cells and enhance the process of angiogenesis. Gnecchi et al. [85] showed that MSCs overexpressing Akt1 enhance myocardial protection post infarction through paracrine mechanisms, reducing apoptosis and improving cardiac function, proposing their secreted factors as potential therapeutic agents for ischemic damage. Kinnaird et al. [86] have shown that MSC secrete arteriogenic cytokines like VEGF and bFGF, enhancing the proliferation of vascular cells through paracrine mechanisms rather than direct incorporation into vessels. In a mouse model, MSC injection improved limb perfusion and functionality and reduced muscle atrophy and fibrosis, underscoring the therapeutic potential of MSCs for collateral remodeling and recovery after ischemic injury [86].

### 3.6. Anti-Fibrotic Effects

Cardiac fibrosis, characterized by the excessive deposition of extracellular matrix (ECM) components, primarily fibrillar collagens like types I and III along with other ECM constituents such as fibronectin and elastin, leads to the stiffening of the heart muscle, impairing its function [87]. MSCs exert anti-fibrotic effects by inhibiting fibroblast proliferation and ECM deposition by promoting the secretion of matrix metalloproteinases, which break down ECM components via cardiac fibroblasts [88]. Kou et al. [89] showed that MSC-derived EVs are effective in modulating immune responses and facilitating the regeneration of various tissues, including those damaged by fibrosis. However, challenges in preparing MSC-EVs, such as ensuring consistent quality and overcoming heterogeneity, are noted as significant hurdles to their clinical application [89]. MSCs release factors such as hepatocyte growth factor (HGF) and prostaglandin E2 (PGE2), which have been shown to directly inhibit the proliferation and activation of fibroblasts into myofibroblasts [88,90]. MSCs directly inhibit the synthesis of TGF-β and also alter downstream SMAD signaling, which is crucial for the transcriptional activation of fibrotic genes [91]. MSC-derived EVs containing microRNAs such as miR-378 and miR-27b have been shown to suppress TGF-β signaling, thereby inhibiting the fibrotic response [92]. Understanding these mechanisms is crucial for developing targeted therapies to treat cardiac fibrosis, a common aftermath of various heart diseases, including myocardial infarction and hypertensive heart disease [93].

## 4. Homing and Migration of MSCs

MSCs have an intrinsic ability to specifically target and migrate to sites of injury or inflammation, such as damaged cardiac tissues, following systemic administration. This process is intricately regulated by a series of molecular signals involving chemokines, their receptors, and adhesion molecules [94]. Chemokines are a family of small cytokines or signaling proteins secreted by cells. The interactions between these chemokines and their receptors on MSCs play pivotal roles in guiding the migration of MSCs to injured heart tissue [95]. Chemokine receptors, such as CXCR4, CCR2, and CCR7, expressed on MSCs allow them to respond to the gradient of chemokines released from the injured or inflamed tissue, a phenomenon akin to a cellular GPS system [95].

Ruster et al. [96] demonstrated the coordinated rolling and adhesion behavior of MSCs on endothelial cells, which is a critical step in the homing process. This interaction is mediated by selectins and their ligands as well as integrins, which facilitate the initial weak binding (rolling) of MSCs on the endothelial surface, followed by firmer adhesion [96]. This adhesion is necessary for MSCs to transmigrate across the endothelial barrier and reach the site of injury [96]. Further studies by Yau et al. [97] have emphasized the roles of specific chemokines, such as SDF-1 (stromal cell-derived factor-1) and its interaction with CXCR4 on MSCs. SDF-1 is upregulated in damaged cardiac tissues and acts as a strong chemoattractant for MSCs, guiding their migration to these sites [97]. In addition to understanding these natural homing mechanisms, research is also focused on how to enhance this ability for therapeutic applications. Approaches such as preconditioning MSCs (with hypoxia or pharmacological agents), genetic modification to overexpress certain chemokine receptors, or even the use of magnetic nanoparticles for guided delivery, are being explored [94]. By enhancing their homing efficiency, it is possible to increase the therapeutic efficacy of MSCs, ensuring a greater number of these cells reach and engraft in damaged tissue, thereby augmenting repair and regeneration processes [94].

### Stimulation of Homing and Recruitment of Stem Cells

The process of stem cell homing and recruitment involves the directed migration and engraftment of stem cells to sites of injury. Recent evidence indicates that chemokine receptor CXCR4 signaling in endothelial progenitor cells is impaired in individuals with coronary artery disease [98]. This impairment leads to reduced neovascularization, highlighting the potential of targeting CXCR4 to improve outcomes in coronary artery disease [98]. Furthermore, the SDF-1/CXCR4 axis has emerged as a promising therapeutic target for ischemic heart disease [99]. Enhancing SDF-1/CXCR4 signaling through various molecular mechanisms, including gene transfer, may augment EPC migration and improve therapeutic efficacy [99].

The development of chemokine-coated scaffolds and the use of nanoparticles sensitive to reactive oxygen species for targeted delivery to injured tissues are innovative approaches being explored [100]. Such nanoparticles have shown promise in targeting CXCR12 in damaged cardiac tissue, potentially enhancing the homing and effectiveness of stem cell therapy [100]. The integration of Artificial Intelligence (AI) in healthcare has opened new avenues for advancing stem cell therapies. AI-assisted drug synthesis and delivery could potentially surpass traditional methods in efficiency and specificity. AI applications in stem cell therapy are poised to deepen our understanding of MSC mechanisms and address existing challenges, thereby enhancing the effectiveness of treatments [101].

Despite over two decades of research and significant advancements, cell-based therapy for cardiovascular diseases faces persistent challenges and uncertainties. Major hurdles include an insufficient number of engrafted stem/progenitor cells, low survival rates in damaged tissue, and the impaired reparative capacity of these cells in patients with cardiovascular diseases [102]. To achieve successful cardiovascular repair through cell-based therapy, these obstacles must be addressed. A safe, effective, and broadly applicable cell-based therapy for cardiovascular diseases necessitates further research and the optimization of stem-/progenitor-cell-based treatments.

## 5. Challenges and Needs in MSC-Based Cardiovascular Therapeutics

The multipotential nature of MSCs is particularly valuable for treating cardiovascular disorders due to their specific differentiation into cell types crucial for cardiovascular therapy [103,104,105].

### 5.1. Challenges

The immunomodulatory capabilities of MSCs are subject of research in treating immunodeficiency diseases, such as graft-versus-host disease and Crohn’s disease. However, the use of MSCs in conditions like multiple sclerosis remains experimental and should be approached with caution [106,107,108]. Despite the potential of MSC-based therapies, challenges have arisen, notably concerning the quality and delivery of MSCs, as well as a lack of established guidelines for culture protocols and biosafety [109,110]. Critical stages in MSC therapy involve the isolation and culture of cells in vitro followed by their in vivo delivery, presenting challenges like impaired homing ability, poor cell retention, and the risk of the overexpression of chemokines and cytokines in targeted areas [45,57,58].

The efficacy of MSCs is also influenced by factors such as the donor’s and recipient’s ages, medical histories, and genetic predispositions [111,112]. Obtaining enough healthy MSCs from individuals with conditions like diabetes or rheumatoid arthritis is particularly challenging as these conditions can affect the quality of MSCs [113]. Additionally, there have been concerns regarding the safety of MSC therapies, such as the development of tumors, which might be attributed to the inherent characteristics of MSCs rather than graft rejection [114].

### 5.2. Overcoming Limitations

To address these challenges, MSCs are now classified as advanced therapy medicinal products. Guidelines from the American Code of Federal Regulations and the Food and Drug Administration, along with GMPs, have been established to guide the culture method, isolation method, quality assurance method, and delivery protocol used and the overall safety of MSC-based therapies [115,116]. These guidelines aim to enhance the in vivo and in vitro efficacy and viability of MSCs. Recent studies, like the one conducted by Codinach et al. [117], have demonstrated the efficacy of bioprocess engineering in the separation, expansion, validation, and manufacture of bone marrow-derived MSCs for clinical use. Their research on 48 batches of iliac crest bone marrow samples for autologous transplantation highlights the importance of standardized processes in MSC therapy, including collection, isolation, trypsinization, and quality control, ensuring the safety, functionality, and potency of MSCs [117].

Adult-tissue-derived MSCs exhibit significant variability due to donor differences, impacting their proliferation, differentiation, and immunomodulatory capacities. This variability challenges the consistency and therapeutic efficacy of MSC-derived products like exosomes. In contrast, iPSC-derived MSCs, which originate from clonal lines, provide a more standardized source that diminishes variability and enhances the scalability and reproducibility of MSC production [118]. These cells are produced under stringent GMP conditions, ensuring rigorous quality control standards for safety, consistency, and efficacy [118]. iPSC-derived MSCs have demonstrated promising results in clinical trials for conditions such as refractory graft-versus-host disease (GVHD), and a Phase 1 trial highlights the potential of iPSC-derived MSCs to surmount challenges of heterogeneity in adult-tissue-derived MSCs, favoring their use in therapeutic applications that require high consistency and scalability [118].

## 6. Emerging Strategies in MSC-Based Tissue Engineering and Regeneration

### 6.1. Cell-Free Approaches: The Power of the Secretome

The MSC secretome, which encompasses a range of bioactive factors, including growth factors, cytokines, and chemokines, contributes to immunomodulation, homeostasis, tissue repair, and regeneration [119,120,121]. The secretome has gained attention in wound healing due to its direct involvement in cell proliferation, migration, and tissue repair [122,123,124]. The process of extracting the secretome from MSCs involves isolating and cultivating the cells in a suitable medium. After appropriate cultivation, the MSC-derived secretome is collected, followed by centrifugation and filtration to ensure purity [125]. Characterization of the secretome proteins is conducted using proteomic techniques such as the shotgun method, ELISA, or Western blotting [126].

Prior to clinical application, the secretome undergoes rigorous testing for efficacy, cell viability, proliferation, wound-healing capabilities [127,128,129,130], and cardiovascular diseases [57,131]. Cell viability assays are conducted using model cells like human epithelial stem cells, and the results are analyzed through fluorescence microscopy. Proliferation assays and colorimetric assessments are also performed to further validate the therapeutic potential of the secretome [127,128,129,130]. The efficacy of the secretome in wound healing is evaluated both in vitro and in vivo [132]. For in vitro studies, tissue defects are treated with varying concentrations of the secretome, and cell migration is assessed using fluorescence analysis [132]. In vivo studies involve applying the secretome to damaged skin in animal models, followed by a histological examination post regeneration [132]. Various delivery methods, including intravenous injection and direct injection into specific tissues, have been explored for secretome administration [133]. While these methods have shown positive effects in animal models, challenges remain, particularly in developing effective culture techniques and standardized protocols for isolation, culture, and distribution to ensure safety and minimize potential complications [133].

The secretome presents a potential therapeutic approach in cell-free treatments which offers advantages such as reductions in the risk of immunological rejection and tumorigenic potential. Its application in tissue regeneration is particularly promising due to its compatibility and ease of delivery, addressing some limitations of traditional stem cell-based therapies. However, further research is needed to establish standardized guidelines and validate its efficacy as a biological element in tissue regeneration [134].

### 6.2. Scaffold-Based Therapeutics: Enhancing Stem Cell Potential

The development of scaffold-based therapeutics represents a significant advance in tissue engineering, offering promising strategies for repairing damaged cardiac tissues and restoring their structure and function [135,136]. These scaffolds, derived from both synthetic and natural biomaterials, are crucial in regenerating diverse tissues including bone, cartilage, ligaments, neural tissues, skin, skeletal muscle, and blood vessels [137,138]. They typically employ biodegradable natural polymers such as collagen, fibrin, gelatin, hyaluronic acid, and poly(lactic-co-glycolic) acid [139,140,141]. The primary objective of biomaterial scaffold-based techniques is to support tissue repair and regeneration. This is achieved by incorporating therapeutic cells into a porous 3D scaffold enriched with growth factors or signaling molecules, creating a conducive environment for cell infiltration, proliferation, and differentiation [142].

#### Biomaterials

Cells, sourced from allogeneic, syngeneic, xenogeneic, or autologous origins, are initially isolated from biopsies. These cells are then cultured in bioreactors, cell culture systems, or in vitro settings for controlled growth and expansion. Expanded cells are seeded onto scaffolds infused with growth factors and nutrients, leading to the formation of new tissues. These tissues, which are integrated within the scaffold, are designed to replace damaged tissues in patients [143,144]. In tissue engineering, natural polymers like chitosan, collagen, alginate, silk fibroin, hyaluronan, and gelatin are extensively used for cartilage regeneration. These materials facilitate the growth of new chondral tissue at defect sites due to their biocompatibility, biodegradability, minimal immune response, and effective cell interaction [145]. Natural polysaccharides, which are preferred over synthetic polymers, potentially minimize immune reactions and promote cartilage growth through specific biological pathways [146,147].

The success of biomaterials in tissue engineering depends on their structural and functional compatibility with target tissues and cell types. This necessity stems from the distinct physical and chemical properties inherent to different tissues and cells [148,149]. Factors such as the hydrophobicity, chemical composition, and charge of biomaterial surfaces significantly influence their biological activity [150,151]. Biomaterials interact intricately with the 3D microenvironments of targeted tissues [152,153,154]. For tissues subjected to mechanical stress or weight bearing, like bones or teeth, biomaterials with robust mechanical properties are essential [155]. Conversely, soft tissues like skin and internal organs require biomaterials with characteristics such as porosity, softness, and high viscosity [156]. This complex interplay between biomaterial properties and tissue-specific needs underscores the critical role of biomaterials in orchestrating successful tissue regeneration processes.

### 6.3. Three-Dimensional Ex Vivo Propagation and Pre-Treatment

The transition from traditional 2D cultures to 3D environments represents a significant advancement in MSC propagation. Three-dimensional cultures better replicate the natural environment of MSCs, enhancing their differentiation toward cardiac [157,158,159] and skeleton-related tissues [160]. Despite extensive research on 3D MSC culture, challenges persist, including replicating the natural ECM milieu, eliminating residual hazardous solvents, achieving uniform cell distribution, and maintaining cell viability [160,161]. Scaffold-based culture systems promote MSC–matrix interaction, while scaffold-free cultures rely on the cells themselves to establish a suitable microenvironment [162]. The growing body of research on 3D MSC culture suggests its therapeutic potential, but it requires more meticulous attention and critical analysis compared to 2D culture. The relative scarcity of standardized protocols for 3D culture, in contrast to the well-established 2D culture methods, presents an additional challenge [163,164].

Optimizing MSC-based treatments involves strategies that do not necessarily rely on increasing the dosage or frequency of administration. The primary goal is to achieve optimal results without adverse systemic effects. These optimization strategies are divided into genetic and non-genetic modifications [165,166]; the latter are also known as MSC pre-treatments. MSCs can be pre-activated to enhance their functional potential by simulating either their physiological or pathological microenvironment [166].

#### 6.3.1. Physiological Microenvironment Simulation Pre-Activation

Typically, in vitro MSC cultures are exposed to an oxygen tension of about 21%. Under hypoxic conditions, MSCs, particularly adipose-derived MSCs, have been shown to maintain their undifferentiated state and express higher levels of multipotent stem cell markers (Oct4, Sox2, and Nanog) without significant morphological or surface marker changes [167,168,169].

#### 6.3.2. Pathological Microenvironment Simulation Pre-Activation

**Figure 1:**
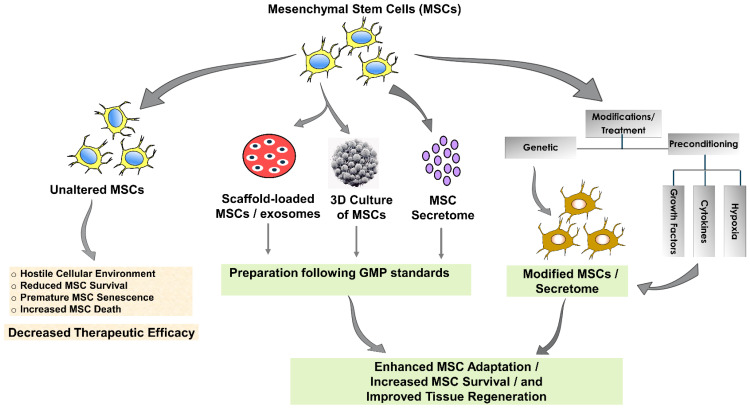
Optimization strategies for enhanced MSC therapeutic efficacy in cardiovascular treatments. This diagram depicts the process of enhancing the efficacy of mesenchymal stem cells (MSCs) for cardiovascular applications. Unmodified MSCs face a hostile cellular environment, leading to reduced survival, premature senescence, and increased cell death, which collectively result in decreased therapeutic efficacy. On the other hand, MSCs undergoing preparation following GMP standards or genetically modified MSCs or MSCs preconditioned with growth factors, cytokines, or hypoxic conditions overcome the limitations of the unmodified state. The resultant modified MSCs exhibit enhanced adaptation to the injury environment, increased survival, and improved capacity for tissue regeneration, thus significantly improving therapeutic outcomes. The figure highlights the critical role of MSC optimization in advancing regenerative cardiovascular medicine. The figure above was partly generated using Servier Medical Art, provided by Servier, licensed under an unported Creative Commons Attribution 3.0 license.

Mimicking the inflammatory microenvironment using cytokines like TNF-α, INF-γ, IL-1β, IL-17A, and IL-25 can enhance the immunomodulatory function of MSCs [170]. Pre-activation with growth factors such as basic fibroblast growth factor (bFGF) and chemokine ligands like SDF-1 has shown promise in maintaining stem cell properties and aiding tissue regeneration [171,172]. Pre-activation with bioactive compounds, either natural or synthetic, in MSC pre-activation is gaining interest [173,174,175,176,177]. These compounds can enhance MSC survival, immunomodulation, and cardiac repair. Notable examples include trimetazidine, tadalafil, atorvastatin for MSC modulation, and iron chelator deferoxamine and treprostinil for enhancing immunomodulatory functions [173,174,175,176,177]. The process of optimizing MSCs for enhanced therapeutic outcomes in cardiovascular treatments has been illustrated in Figure 1.

### 6.4. Genetic Modifications: Tailoring MSCs for Targeted Repair

Genetic modifications of MSCs for targeted repair are performed using both viral vectors and non-viral methods, enhancing specificity and efficacy in regenerative treatments. The ease of isolation and expansion of MSCs in vitro makes them suitable candidates for transfection and targeted recruitment at inflammation sites [178,179,180].

#### 6.4.1. Viral Vector-Mediated Genetic Modification

Retroviruses, using RNA as genetic material, integrate their genome into the host cell DNA [181]. This process involves the fusion of the viral lipid envelope with the host cell membrane, followed by the reverse transcription of viral RNA into DNA and its integration into the host genome [181]. Retroviruses have been used to modify MSCs efficiently, allowing for the production of large quantities of modified cells [182]. For example, a study successfully transferred genes like Foxa1 and Hnf4a into BMSCs using retroviral methods [183]. Lentiviruses, which are capable of infecting non-dividing cells, can carry 8–9 kb of genetic material. Unlike retroviruses, lentiviruses integrate more slowly and less disruptively into the host cell genome. Their high efficiency and stability rates make them preferred tools for MSC modification [184,185,186]. The transplantation of MSCs modified with the TNFR gene via a recombinant adeno-associated virus improved left ventricular function after myocardial infarction by reducing inflammation and apoptosis [187].

#### 6.4.2. Non-Viral Methods of Genetic Modification

Electroporation, sonotransfection, and nucleofection are physical methods for introducing genetic material into MSCs [188,189]. Electroporation has shown high efficiency, with the optimal conditions for human MSCs being a pulse magnitude of 1500 V for 20 ms, resulting in 78% viability and 50% efficiency [188]. Nucleofection, a specialized form of electroporation, facilitates the direct transfer of plasmid DNA into the cell nucleus [188]. Chemical methods include the use of synthetic vectors like cationic lipids, polymers, nanoparticles, and cell-penetrating peptides for gene transfer [190]. Chemical methods offer the advantage of large-scale manufacturing and reduced side effects compared to viral vectors [190]. Genetically engineered MSCs overexpressing stromal cell-derived factor-1α [191] or hypoxia-inducible factor 1-α [192], Insulin-like growth factor 1, and hepatocyte growth factor [193] have shown potential in cardiovascular recovery. MSCs modified to express specific factors demonstrated enhanced efficacy in reducing inflammatory responses and fibrosis in a model of Chagas disease [194]. Additionally, MSCs overexpressing microRNA, such as miR-126, have been shown to increase pro-angiogenic factors, thus improving the treatment efficacy in infarcted hearts [195].

### 6.5. Mechanobiologically Mediated Differentiation of Stem Cells

Shear stress from fluid flow crucially directs MSCs and EPCs toward an endothelial phenotype, elevating endothelial markers like CD31, vWF, and vascular endothelial cadherin due to mechanotransduction pathways that translate mechanical signals into biochemical cues [54]. The application of laminar shear stress to MSCs significantly increased cardiomyocyte differentiation [196] and endothelial markers, promoting endothelium-like functions such as tubule formation and low density lipoprotein uptake [197]. Additionally, mechanical strains, such as cyclic stretch, also guide stem cells toward endothelial lineages, particularly in EPCs, enhancing their migration and tubulogenesis, which mirrors the dynamics within blood vessels [198]. This integration of mechanobiological stimuli is essential for the effective differentiation of MSCs and EPCs into functional ECs, underscoring the significance of physical cues in vascular tissue engineering and the potential of these cells in regenerative medicine for cardiovascular disorders [27,54]. The optimization of cell culture conditions, microenvironmental factors, and the application of mechanical and electrical stimulations to enhance myogenic differentiation has been emphasized [199,200].

A comparative overview of emerging MSC-based therapeutic strategies in tissue engineering and regeneration is summarized in Table 1.

### 6.6. GMPs in Stem Cell-Based Therapeutics

MSCs must comply with current GMP standards, ensuring their readiness for further manufacturing steps. GMPs, enforced by agencies like the US Food and Drug Administration (FDA), set forth rigorous rules to guarantee that products are consistently produced and controlled according to quality standards [201]. Adherence to GMP standards is a fundamental requirement for obtaining marketing authorization for stem cell-based therapeutics [202]. This compliance not only accelerates the development of regenerative medicine products but also potentially reduces associated costs [203]. Global regulations and quality assurance guidelines provide detailed procedures for manufacturing, testing, and quality assurance. Adhering to these standards is crucial for producing raw materials that meet stringent criteria for purity, potency, consistency, and stability [203]. Compliance with these guidelines is vital for maintaining the safety and high quality of raw materials used in stem cell-based therapies, thereby upholding the integrity and efficacy of the final therapeutic products [203].

## 7. Conclusions

Emerging strategies in MSC-based cardiovascular therapeutics focus on sophisticated, targeted approaches that move beyond traditional cell transplantation to more refined treatments. Harnessing the MSC secretome, developing biocompatible scaffolds, employing genetic modifications, and utilizing extracellular vesicles and three-dimensional tissue modeling are key advances. These strategies aim for higher efficacy, reduced side effects, and personalized patient care. The integration of tissue engineering, nanotechnology, and precision medicine is set to enhance these therapies further, ensuring their safety, quality, and reproducibility through standardized protocols and GMP-compliant procedures. This evolving landscape promises transformative impacts on cardiovascular medicine, driven by in-depth MSC biology research and technological innovations.

## Figures and Tables

**Table 1:** Overview of emerging MSC-based therapeutic strategies in tissue engineering and regeneration.

Method	Feasibility	Advantages	Disadvantages	References
Cell-Free Approach (Secretome)	The MSC secretome encompasses a range of bioactive factors, including growth factors, cytokines, and chemokines, which contribute to cell proliferation, migration, and tissue repair.	1. Risk of immunological rejection is minimized; 2. Secretome offers a cell-free treatment option; 3. High compatibility with host tissues; 4. Ease of delivery; 5. Lower tumorigenic potential compared to cellular therapies.	Limited by the absence of standardized protocols for secretome preparation	[57,121,122,123,124,125,131]
Scaffold-based Therapeutics	Scaffold-based treatments have emerged as a notable breakthrough in tissue engineering, providing promising approaches for healing injured tissues and reinstating their structure and function.	Essential for regenerating a variety of tissues, providing structural support and a conducive environment for cell attachment and growth.	Potential for host versus graft rejection and suboptimal mechanical properties that may not withstand long-term stress	[135,136,137,138,139,140,141]
Three-Dimensional Ex Vivo Propagation	The aggregation of MSCs in a three-dimensional (3D) structure enhanced several biological characteristics, such as the ability to differentiate into multiple cell lineages, the production of therapeutic factors, and the ability to withstand ischemic conditions.	Enhances differentiation toward skeleton-related tissues and the production of therapeutic factors.	Challenges include replicating the ECM, maintaining uniform cell distribution, and ensuring high cell viability	[157,158,159,160,161]
Physiological and Pathological Microenvironment Activation	Exposing MSCs to varying oxygen tensions and inflammatory cytokines simulates physiological and pathological conditions, respectively.	Physiological activation maintains stemness under hypoxia; pathological activation enhances immunomodulation and tissue regeneration responses via cytokines and growth factors.	Precise environmental control is needed to simulate conditions effectively, posing operational challenges	[167,168,169,170,171,172,173,174,175,176,177]
Genetic Modification	Genetic modifications of MSCs for targeted repair are performed using both viral vectors and non-viral methods to enhance specificity and efficacy.	1. High efficiency and stability in viral gene delivery; 2. Reduced inflammatory responses and fibrosis in disease models; 3. Boosted therapeutic effectiveness for infarcted hearts with miR-126.	Side effects from viral-mediated delivery and high costs	[184,185,186,187,191,192,193]
Mechanobiology-Mediated Differentiation	Shear stress and mechanical strains direct MSCs and EPCs toward an endothelial phenotype, enhancing endothelial markers and functions.	1. Promotes endothelial-like functions, enhancing tubule formation and LDL uptake; 2. Guides stem cells toward endothelial lineages, enhancing migration and tubulogenesis, which are crucial for vascular tissue engineering.	Requires precise control over mechanical conditions to ensure effective differentiation and functional outcomes in ECs	[54,196,197,198,199,200]
